# Applications of indocyanine greenenhanced fluorescence in the laparoscopic treatment of colonic stricture after necrotizing enterocolitis

**DOI:** 10.1186/s12887-023-04458-4

**Published:** 2023-12-16

**Authors:** Xiaohong Die, Mengying Cui, Wei Feng, Jinfeng Hou, Pengfei Chen, Wei Liu, Fang Wu, Zhenhua Guo

**Affiliations:** Department of General & Neonatal Surgery, National Clinical Research Center for Child Health and Disorders, Ministry of Education Key Laboratory of Child Development and Disorders, Chongqing Key Laboratory of Pediatrics, Children’s Hospital of Chongqing Medical University, Chongqing, China

**Keywords:** Indocyanine green, Laparoscopic, Necrotizing enterocolitis, Colonic stricture

## Abstract

**Background:**

The status of anastomotic blood perfusion is associated with the occurrence of anastomotic leakage after intestinal anastomosis. Fluorescence angiography (FA) with indocyanine green (ICG) can objectively assess intestinal blood perfusion. This study aims to investigate whether anastomotic perfusion assessment with ICG influences surgical decision-making during laparoscopic intestinal resection and primary anastomosis for colonic stricture after necrotizing enterocolitis.

**Methods:**

Patients who underwent laparoscopic intestinal resection and primary anastomosis between January 2022 and December 2022 were retrospectively analyzed. Before intestinal anastomosis, the ICG fluorescence technology was used to evaluate the blood perfusion of intestinal tubes on both sides of the anastomosis. After the completion of primary anastomosis, the anastomotic blood perfusion was assessed again.

**Results:**

Of the 13 cases, laparoscopy was used to determine the extent of the diseased bowel to be excised, and the normal bowel was preserved for anastomosis. The anastomosis was established under the guidance of ICG fluorescence technology, and FA was performed after anastomosis to confirm good blood flow in the proximal bowel. The anastomotic intestinal tube was changed in one case because FA showed a difference between the normal range of intestinal blood flow and the macroscopic prediction. There was no evidence of ICG allergy, anastomotic leakage, anastomotic stricture, or other complications. The median follow-up was 6 months, and all patients recovered well.

**Conclusions:**

The ICG fluorescence technology is helpful in precisely and efficiently determining the anastomotic intestinal blood flow during stricture resection and in avoiding anastomotic leakage caused by poor anastomotic intestinal blood flow to some extent, with satisfactory short-term efficacy.

## Introduction

Necrotizing enterocolitis (NEC), a common acute abdominal infectious disease, has a high prevalence in premature and low birth weight neonates [1]. With the improvement of neonatal treatment technology in recent years, the mortality rate of this disease has decreased yearly, but the incidence of NEC treatment-related complications has increased [2]. Colonic stricture, a common complication of NEC, has an incidence ranging from 11.0 to 35.0% [3, 4]. Surgery is the only effective way for colonic stricture after NEC, and the mainstream surgical method was stenotic intestinal resection and primary anastomosis. Laparoscopy, a development of endoscopy technology, is frequently used to treat colonic stricture after NEC [5].

Intraoperative strictures can be surgically resected under laparoscopic guidance, which prevents postoperative anastomotic leakage and improves the prognosis. Anastomotic leakage, in particular, is directly related to a poor prognosis and death in neonates, which increases the length and cost of hospitalization [6]. There is no clear data on the annual incidence of anastomotic leakage in infants following anastomosis resection for colonic strictures. In children with Hirschsprung disease, the rate of anastomotic leakage after pull-through procurement is 5-7% [7, 8], and the rate of anastomotic leakage after colorectal cancer surgery in adults is 3-20% [9]. The pathogenesis of anastomotic leakage is still unknown. Previous research has shown that the condition of anastomotic blood perfusion is the most important factor influencing the outcome of anastomotic healing [10]. At present, the assessment of anastomotic blood perfusion is primarily based on the clinical experience of surgeon, which is somewhat subjective. However, some studies have suggested that the operating surgeon’s clinical judgment was based on the underestimated risk of anastomotic leakage in colorectal surgery [9].

Fluorescence angiography (FA) with indocyanine green (ICG) is a new technique used in adult gastrointestinal surgery to reduce the incidence of anastomotic leakage [11]. Intraoperative blood perfusion at the colonic anastomosis can be assessed using an FA imaging system, and the surgeon can perform intestinal anastomosis based on the perfusion or change in the scope of excision. A recent systematic review demonstrated that using FA with ICG for pediatric intestinal resections is feasible and safe [12]. In addition, FA with ICG may affect intestinal resection margins and potentially shorten the extent of resection in pediatric surgery [13].

In the current report, we examine the outcomes of patients with colonic stricture after NEC who were diagnosed and successfully treated with laparoscopic intestinal resection and primary anastomosis using ICG-enhanced fluorescence. Additionally, real-time ICG angiography was employed to identify the blood perfusion of anastomosis site and ensure complete resection of the stricture segment. This report is the first to describe this strategy in the laparoscopic treatment of colonic stricture after NEC using FA with ICG.

## Materials and methods

### Patients

The clinical characteristics of patients with colonic strictures admitted to the Children’s Hospital of Chongqing Medical University between January 2022 and December 2022 were retrospectively collected. Inclusion criteria: a clear history of NEC and conservative treatment with hospitalization; clinical manifestations including recurrent abdominal distension, vomiting, bloody stools, and feeding intolerance; and intestinal strictures identified by pathological testing and intraoperative investigation. Exclusion criteria: patients who had a gastrointestinal perforation or were in critical condition and needed an emergency fistulotomy; patients with a known allergy or sensitivity to iodine; patients with severe cardiac or pulmonary disease; and patients whose guardians did not sign the informed consent form.

This study was approved by the ethics committee of the Children’s Hospital of Chongqing Medical University (2022 − 257). All procedures in the study were carried out in accordance with national ethical guidelines for medical and health research involving human subjects, as well as the 1964 Helsinki Declaration and its subsequent amendments.

### ICG

ICG is a water-soluble tricarbocyanine dye with a high affinity for plasma protein in blood vessels and an excellent ability to enter the vascular system and tissues. When illuminated by excitation light with a wavelength of 760 nm, ICG can produce near-infrared light with a wavelength of 820 nm. Its signals can also be detected in deep [14]. ICG fluorescence imaging has high sensitivity and allows deep penetration for easy detection without the use of ionizing radiation, which is used in traditional imaging methods [15]. The use of FA with ICG in pediatric surgery is acceptable, feasible, and safe, according to previous research [12]. In particular, FA with ICG, as opposed to conventional visual assessment, allows modification of the location parameters and the extent of bowel resection. The ICG used in this study was 25 mg per dose (Dandong Medical Innovation Pharmaceutical Co., LTD., Approval number: H20055881).

### Laparoscopic system

The HyPixelTM R1 (Shenzhen Mindray Bio-Medical Electronics Co., Ltd.) A 4 K endoscopic fluorescence camera system was used in this study, and the probe could switch between white light and ICGF mode at any time. Under the guidance of this device, all cases underwent intestinal stricture resection and anastomosis.

### Procedure

The patient underwent the same procedure as with traditional laparoscopic exploration. The suspected lesion area was first investigated based on the stricture site identified by preoperative barium enema contrast. After locating the lesion, the remaining colon and ileum were thoroughly examined to rule out multiple intestinal strictures. Before the diseased intestine was separated, 25 mg ICG was dissolved in 10 ml sterilized water (2.5 mg/ml) for injection. The appropriate amount of ICG was administered intravenously based on the patient’s weight. Fluorescence laparoscopy was used to assess the diseased intestine and blood flow of the distal and proximal intestinal tubes following narrow intestine resection. Following primary anastomosis, the same dose of ICG was injected into a peripheral vein to allow fluorescence imaging of the anastomosis and evaluate its blood supply.

## Results

### Patient characteristics

The study included thirteen patients (Table 1), including nine males and four females. The median weight was 3.17 (2.34,5.21) kg, and the median age was 60 (36,90) days. In addition, the blood type and ASA score were recorded (Table 1).

**Table 1:** Patient characteristics

Characteristics	N = 13
Gestational age (weeks)	34.4 ± 3.1
Age at surgery (days)	60 (36,90)
Birth weight (kg)	2.28 (1.79,3.38)
Weight at surgery (kg)	3.17 (2.34,5.21)
Sex, n(%)	
Male	9 (69.23%)
Female	4 (30.77%)
Blood type, n(%)	
A	4 (30.77%)
B	2 (15.38%)
AB	0
O	7 (53.85%)
Anesthetic risk	
ASA I	0
ASA II	4 (30.77%)
ASA III	9 (69.23%)
ASA IV	0
NEC Bell’s stage	
IIa	8 (61.54%)
IIb	5 (38.46%)

Data are presented as mean ± standard deviation, median (IQR, interquartile range) or frequency (%)

### Operative details and outcomes

**Fig. 1:**
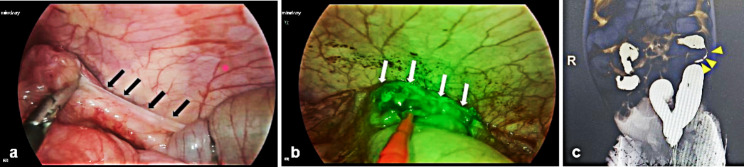
Colon stenosis sites are characterized by ICG accumulation. **(a)** Laparoscopic exploration of stenosis in the descending colon (black long arrow indicates the stricture segment of the colon). **(b)** Green fluorescence accumulation was observed in the stricture colon three minutes after intravenous ICG administration (white long arrow indicates the stricture segment of the colon). **(c)** Stricture in the descending colon was shown by barium contrast (yellow short arrow indicates the stricture segment of the colon)

Thirteen patients underwent ICG fluorescence imaging. There were no ICG-related adverse events, such as rash, fever, or anaphylactic shock, in any of the patients. The time required to achieve sufficient ICG fluorescence imaging of the colon was 20 (15, 40) seconds, and the total duration was 4 (3, 5) minutes (Table 2). The first patient in this study weighed 3.5 kg, and the ICG dose was 2.5 mg (0.714 mg/kg). It was discovered that the fluorescence intensity was too high and the contrast was poor, making judging the local blood perfusion difficult. Starting with the second patient, we reduced the amount of ICG based on the patient’s weight and captured a clear ICG fluorescence image (Table 3). Our results suggest that ICG accumulation in the colon alerts the site of the stricture and results in slow fluorescence fading (Fig. 1). Local necrosis and lumen stricture in acute NEC of the intestine, as well as local capillary compensatory hyperplasia during the repair period, are thought to be contributing factors [16, 17]. As a result, fluorescence can improve the clarity of the preoperative angiography image so that the site of hidden intestinal stricture can be recognized.

**Table 2:** Operative details and outcomes

Operative details and outcomes	N = 13
Duration of surgery (minutes)	210 [140,260]
Hemorrhage during operation (ml)	10 [5,40]
Multiple intestinal strictures, n(%)	4 (30.77%)
Time to suficient fluorescence (seconds)	20 [15,40]
Duration of fluorescence (minutes)	4 [3,5]
Surgical plan modification with ICG-FA, n(%)	1 (7.69%)
Start of oral feeds postoperatively (days)	5 [3,7]
LOS (days)	9 [7,15]
Complications	
Wound infection, n(%)	1 (7.69%)
Acute intestinal obstruction	0
Postoperative anastomotic stricture	0
Postoperative anastomotic hemorrhage	0
Postoperative anastomotic leakage	0
30-day unplanned rehospitalization,n(%)	1 (7.69%)
Follow-up time after discharge (months)	6[3,9]

LOS: length of hospital stay

Data are presented as median [IQR, interquartile range] and frequency (%)

**Table 3:** Indocyanine green dose administered

Patient Weight	ICG dose per bolus	Maximum ICG dose per procedure
≤ 2.5 kg	0.625 mg/kg	1.875 mg/kg
2.5-4.0 kg	1.25 mg	3.75 mg
≥ 4.0 kg	2.5 mg	7.5 mg

**Fig. 2:**
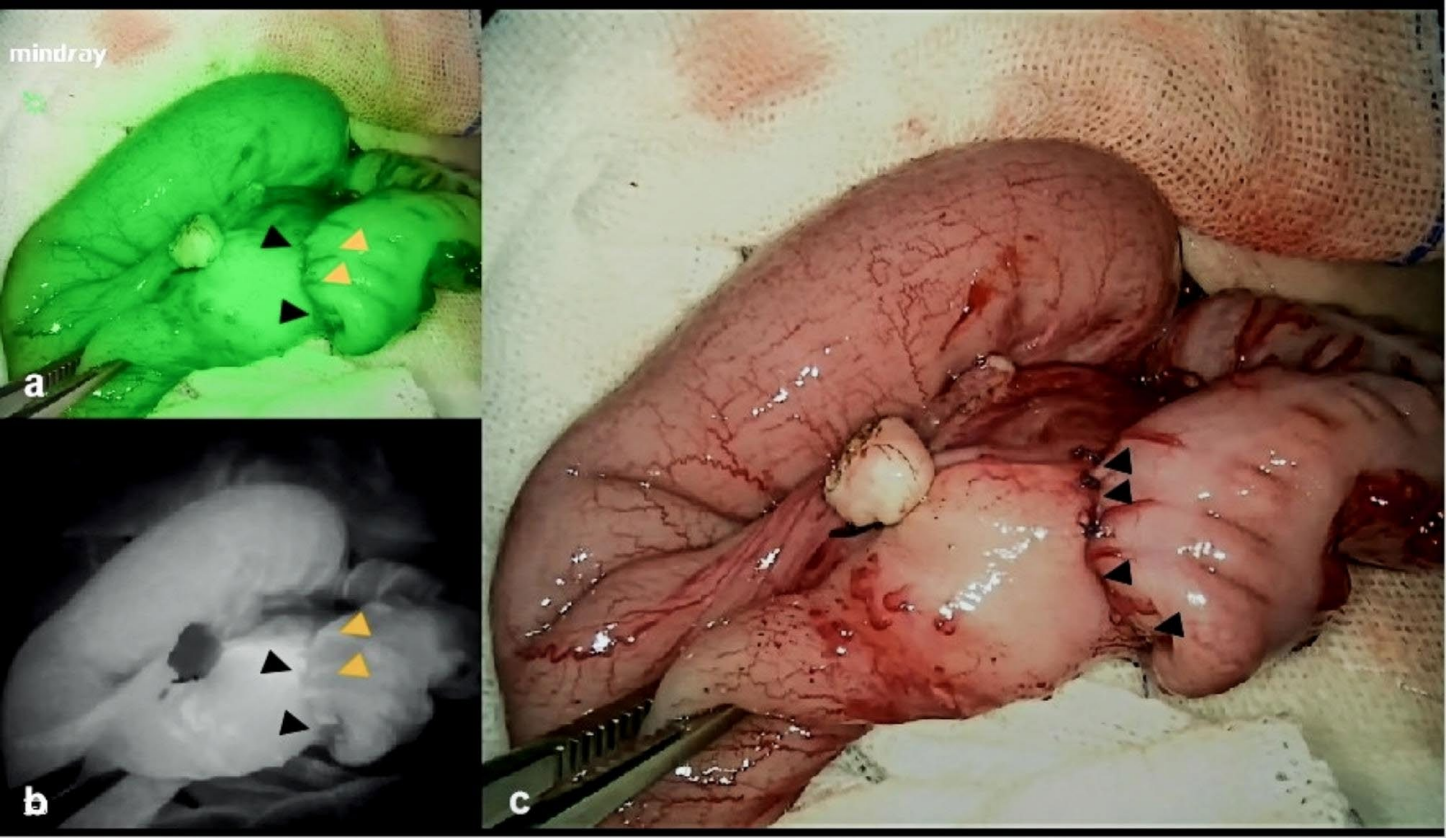
Image of a typical well-perfused anastomosis under ICG fluorescence angiography. **a** and **b** show perfusion under fluorescence angiography with ICG in the colon; **c** shows the anastomosis established in colectomy (black short arrow indicates the anastomosis, yellow short arrow indicates the blood vessels)

After resection of the stricture segment of colon, all patients were judged to have good blood supply, and the blood perfusion of the intestine to be anastomosed was observed under white light. Green fluorescence imaging was visible in the distal bowel of all patients after the ICG injection in fluorescence mode. In 12 patients, proximal bowel imaging was good, but the proximal bowel wall of 1 patient did not show obvious green fluorescence, indicating poor intestinal blood flow and proximal intestinal enlargement excision. ICG was injected again after the anastomosis, and strong fluorescence was seen in both the distal and proximal intestinal tubes of the anastomosis (Fig. 2). Multiple colonic stricture was discovered in four cases (30.77%), but small intestine stricture was not discovered. The median operative time was 210 (140,260) minutes, with a blood loss of 10 (5,40) ml. The median length of postoperative oral feeding was 5 (3,7) days, and that of the hospital stay was 9 (7,15) days (Table 2).

One patient developed an infection in the umbilical incision four days after surgery and recovered after an active dressing change. Acute intestinal obstruction, anastomotic hemorrhage, anastomotic stricture, anastomotic leakage, and other serious complications were not observed. One patient was readmitted to the hospital 25 days after surgery due to adhesive ileus. This patient was admitted and received conservative care, including fasting, gastrointestinal decompression, and fluid rehydration. Five days later, the patient was released. During outpatient follow-up for 6 months, there was no recurrence. The median follow-up time for all patients was 6(3,9) months, with a favorable prognosis.

## Discussion

The most common complication in infants with NEC is intestinal stricture, which mostly affects the colon. Multiple strictures may occur in a few cases and usually require surgical treatment [18]. Late complications include cholestasis, short bowel syndrome, and brain injury, all of which have a serious effect on an infant’s quality of life^[19]^. All patients in this group had colonic stricture, and the incidence of multiple stenoses was 30.77% (4/13). The prognosis was good after postoperative outpatient follow-up.

Surgery is the only effective treatment for intestinal stricture in patients with NEC^[20]^. In most hospitals, intestinal resection and primary anastomosis are performed, and an intestinal biopsy is required to rule out Hirschsprung’s disease. In some medical institutions, end-to-end enterostomy was performed by resecting the narrow segment, protective enterostomy was performed at the proximal end, and enterostomy was performed in stage II. Most institutes now strongly advise one-stage intestinal resection and anastomosis. With the advancement of laparoscopic technology, laparoscopic exploration is now possible in infants [5].

Anastomotic leakage is one of the most serious complications following colectomy and anastomosis, and it can result in serious complications such as pelvic abscess, peritonitis, septic shock, and even death [6]. The incidence of postoperative anastomotic leakage in patients with Hirschsprung’s disease [7, 8] and colon cancer [9] is 10%, according to earlier studies. The incidence of anastomotic leakage has decreased as surgical techniques have improved, but it cannot be completely avoided. Patients with anastomotic leakage had significantly higher in-hospital mortality, longer hospital stays, and higher medical expenses, all of which harmed patients’ short- and long-term prognoses [19]. Anastomotic leakage is caused by the combined action of several factors. Maintaining a tension-free and adequate blood supply to the anastomotic stoma is critical for preventing anastomotic leakage. There is currently no objective quantitative index for assessing anastomotic blood supply, which is primarily determined by the surgeon’s observation of the color of anastomotic intestine, blood oozing at the broken end, and capillary filling [20]. The majority of these examination methods are based on subjective judgments. Even the most experienced operators can make mistakes. Previous research has shown that judging anastomotic blood supply by subjective perception has low sensitivity and specificity [21].

ICG fluorescence imaging is a quick and easy way to assess intestinal blood flow [22]. ICG can quickly bind to plasma protein and flow to mesenteric blood vessels after intravenous injection. It can emit fluorescence after being exposed to near-infrared light, and it can be converted into black-and-white or color images after being absorbed by the probe of a fluorescence imaging system to assess intestinal blood perfusion [23]. ICG was first used in colorectal cancer surgery in 2010 to assess the safety and dependability of anastomotic blood flow [24]. Currently, ICG fluorescence in vascular imaging is widely used in the operation of adult colorectal cancer to judge the blood flow of the anastomotic intestinal canal, which can effectively reduce the incidence of anastomotic leakage [25, 26]. Iinuma et al. [27] reported that this technique was first used in a child to judge intestinal blood flow in a child with intestinal volvulus complicated by intestinal necrosis and that this technique provided more useful data on intestinal blood flow promptly than traditional clinical evaluation. According to a recent prospective study, the use of FA with ICG for pediatric intestinal resections is feasible and safe, and this fluorescence imaging technology may be valuable for intestinal resections in pediatric surgery [12]. The color of the intestinal canal at the anastomotic site was normal in 1 patient (1/13) in this study, but the scope of surgical resection was changed due to poor local imaging after ICG injection. The patient recovered quickly after the operation, and there was no anastomotic leakage. It should be noted that ICG is only observed in specific areas, but did not clearly show blood vessels in our figures, which differs from other references reported. Unlike what has been reported in other references, the site observed in this study is the colon, which itself supplies blood from the terminal branch of the mesenteric vessel and is usually too small to be showed. Furthermore, this study discovered that the slow metabolism of ICG in the stricture can be used to judge the stricture, particularly in occult stricture where preoperative radiography was inconclusive. Furthermore, we found slow fluorescence fading after ICG accumulation, laterally confirming that the stricture site, although had local capillary compensatory hyperplasia, was obstructed in blood reflux. Therefore, the use of ICG to assess the blood perfusion at the stricture site should not only focus on the accumulation of ICG, but also its fading.

This study still has some limitations. This is a single-center study with a small sample size and a short follow-up period. Although recent studies have shown that ICG fluorescence imaging can help reduce the incidence of anastomotic leakage, the determination of fluorescence intensity is still based on surgeons’ visual judgment. As a result, a multicentre, large-sample randomized controlled study is required to determine how to quantitatively measure fluorescence intensity, determine the fluorescence threshold for imaging of intestinal blood perfusion disorder, and establish the standard of ICG fluorescence imaging.

## Conclusion

It is safe to use ICG fluorescence imaging in the resection and anastomosis of colonic Stricture in infants. ICG fluorescence imaging can be used to determine the location of a colonic stricture and the blood flow status of anastomosis, improve the safety of intestinal stricture resection and anastomosis, and reduce the incidence of anastomotic leakage.

## Data Availability

The datasets generated and analyzed during the current study are not publicly available due to the ongoing analysis in other directions but are available from the corresponding author (Zhenhua Guo) on reasonable request.
